# Splenic abscess after laparoscopic sleeve gastrectomy. A rare complication—case report and literature review

**DOI:** 10.1093/jscr/rjab042

**Published:** 2021-04-29

**Authors:** Esam Batayyah, Waed Yaseen, Faris Alshareef

**Affiliations:** General Surgery Department, Alnoor Specialist Hospital, Makkah, Saudi Arabia; General Surgery Department, Alnoor Specialist Hospital, Makkah, Saudi Arabia; General Surgery Department, Asser Central Hospital, Abha, Saudi Arabia

## Abstract

Laparoscopic sleeve gastrectomy is currently a stand-alone bariatric procedure with a low complication profile. A rare complication of leak following sleeve gastrectomy was reported in this study. Its rareness and nonspecific clinical presentation could make the diagnosis difficult and could be easily confused with leak and subdiaphragmatic abscess. A 22-year-old Saudi female with body mass index 41 underwent laparoscopic sleeve gastrectomy in 2017, presented 18 months later to emergency department complaining of fever and abdominal pain for 3 months prior to presentation. Computed tomography of abdomen revealed a large splenic abscess, upper gastrointestinal studies were unremarkable. Patient was taken for laparoscopic exploration with finding of splenic abscess and gastric fistula, splenectomy and clipping of fistula was performed. The management of splenic abscess remains controversial. Splenectomy and antibiotics have generally been the definitive treatment particularly with large multilobulated collection. Familiarity with the rare complications as splenic abscess will allow for a prompt diagnosis and treatment.

## INTRODUCTION

Laparoscopic sleeve gastrectomy (LSG) is now a stand-alone bariatric procedure for morbid obesity with a low complication profile. Some of the reported complications include bleeding, obstruction, leak and wound or intra-abdominal infection [1]. The formation of splenic abscess is an extremely rare consequence of LSG described in three case reports. Its rareness and nonspecific clinical presentation could make the diagnosis difficult and could be easily confused with leak and subdiaphragmatic abscess. We report this case of splenic abscess formation after laparoscopic sleeve gastrectomy.

## CASE PRESENTATION

A 22-year-old Saudi female with a body mass index of 41 and weighing 115 Kilogram (Kg) underwent laparoscopic sleeve gastrectomy (1438 Arabian Higri Calendar). She had a smooth recovery with discharge home on the second postoperative day.

One-month later the patient presented to emergency department (ED) with abdominal pain, vomiting and intolerance to oral intake. Her laboratory workups were within normal, computed tomography (CT) abdomen with oral and intravenous (IV) contrast revealed a collection small 2 × 2 cm with small air pocket adjacent to the staple line at the upper stomach. Patient was treated as leak conservatively, kept NPO on TPN for 4 weeks, her condition improved and she was discharged home.

One-year later she presented to the ED complaining of fever and abdominal pain. Her laboratory workups were within normal. Abdomen CT scan showed a splenic abscess, she was investigated for a leak or fistula, it was observed negative so she was treated with IV antibiotics and then follow-up CT showed complete resolution. She was discharged home.

Three months later patient came to our ED with same presentation and again all investigations radiological and upper gastrointestinal (UGI) endoscopy was negative. The decision was made to take patient for laparoscopic exploration where we found pyogenic membrane all over the abdomen with splenic abscesses and subphrenic collection, all pus in abdominal cavity was suctioned out and splenectomy was completed and drains were left in left subphrenic plane. On the Day 3 post-operatively, the drain showed gastric content, upper GI endoscopy revealed small pinhole fistula, which was cauterized and clip was applied. Patient was placed NPO on TPN for 2 weeks, then resume oral intake gradually and discharged after full recovery.

Follow up 1 month and 6 month and one-year patient were doing will and her condition is stable with last weight 67 KG.

## DISCUSSION

Splenic abscess is a rare and life-threatening condition with an incidence ranging from 0.1 to 0.7% in various autopsy series. It was reported after some gastric procedures like Nissen fundoplication and gastrectomy for cancer. It is also because of the sequela of post-LSG leak and formation of a splenic abscess, as it was reported in 13 cases in English literature [2–10].

Splenic abscess formation was a result of iatrogenic splenic injury during surgery, splenic ischemia after LSG, extension from a gastric staple-line leak and temporary immune suppression in the immediate postoperative course as described by the previous authors [2–10].

In the current case, the splenic abscess was formed as a result of staple line leak, as described in the upper gastrointestinal (UGI) endoscopy pinpoint fistula found in the post-operative endoscopy, which was closed with cattery and clipping followed by keeping patient NPO on TPN for 4 weeks.

In majority of reported cases patients presented with fever, increase leucocytic count and abdominal pain. Our patient experienced similar symptoms. CT scan of abdomen with IV contrast is the golden slandered [7], for diagnosing the splenic abscess was used in our case as well.

Single, non-loculated, small spleen abscesses are treated with IV antibiotics but with larger, multiloculated or multiple abscesses percutaneous or laparoscopic drainage is next step in management with the IV antibiotics to preserve the spleen. When symptoms persist or when multiple abscesses exist, splenectomy remains the definitive management [10]. Our patient initially had a small collection that responded to conservative management with IV antibiotics but after she came back for the third we decided to take her for laparoscopic exploration and splenectomy.

## CONCLUSION

Splenic abscess is a very rare complication after LSG. The mechanism of formation of splenic abscess needs further studies. The patient must benefit from a conservative treatment based on antibiotics and percutaneous drainage. The decision to undergo a splenectomy will be based on clinical status and response to treatment.

## CONFLICT OF INTEREST STATEMENT

None declared.

## FUNDING

None.

## Supplementary Material

spleen_abcsess_rjab042
